# 

**Published:** 1887-12

**Authors:** 


### CONTENTS.

***MISCELLANEOUS.***

| | PAGE |
|---|---|
| The Year's Work, | 439 |
| The Curse of Opiates: A Lay Opinion, | 440 |
| The Law of the Similars the *only* Law of Cure. Ad. Lippe, M. D., | 441 |
| Notes on Characteristics. C. Carleton Smith, M. D., and Edmund J. Lee, M. D., | 443 |
| Lecture upon Sulphur. Professor Kent, | 452 |
| Ailanthus. A. McNeil, M. D., | 456 |
| Irregular Menses. Ad. Lippe, M. D., | 457 |
| Class-Room Talks. Professor Kent, | 458 |
| Materia Medica: What is Needed. S. L., | 460 |
| Aggravation from Noise. S. L. G. Leggett, | 461 |
| Sound and Noise, | 461 |
| A Sabadilla Case. J. Field Deck, M. D., | 463 |
| The Central New York Homœopathic Medical Society, | 466 |
| Chronic Diseases. J. G. Gundlach, M. D., | 474 |
| In Memoriam—Wm. M. Zerns, M. D., | 476 |
| In Memoriam—John K. Lee, M. D., | 476 |
| Soft Corns Cured by Wiesbaden. E. W. Berridge, M. D., | 477 |
| Clinical Cases. J. A. Biegler, M. D., | 477 |
| Hahnemannian Association of Pennsylvania; Report of Proceedings, | 479 |
| Spongia Tosta. C. Carleton Smith, M. D., | 482 |
| Repertory to Hæmorrhage of Bowels. J. V. Allen, M. D., | 485 |
| Typhoid Fever. J. V. Allen, M. D., | 487 |

***BOOK NOTICES AND REVIEWS.***

| | |
|---|---|
| Rimedii Individualizzati del Dottor Tommaso Cigliano, | 488 |
| Principio Obbietto della Materia Medica. Del Dottor Tommaso Cigliano, | 489 |
| Lectures upon Clinical Materia Medica. Professor E. A. Farrington, M. D., | 489 |
| Drugs and Medicines of North America. J. U. & C. G. Lloyd, | 489 |
| How to Study Materia Medica. Conrad Wesselhœft, M. D., | 490 |
| Otis Clapp & Son's Visiting List, | 490 |
| Lindsay & Blakiston's Visiting List, | 490 |
| Books and Pamphlets Received, | 491 |

***NOTES AND NOTICES.***

| | |
|---|---|
| Married—Antipathies—Errata—Early Use of the Magnet in Surgery—Old Style—A Rara Avis—Worms in Eggs—The Ladies' Aid Association—Southern Homœopathic Medical Association—New Homœopathic Dispensary—Give Nature a Chance—Index to Volume Seventh—A Subscriber's Opinion, | 491 |

